# Supplementary figures and images for: Identification of omega-3 oxylipins in human milk-derived extracellular vesicles with pro-resolutive actions in gastrointestinal inflammation
Source: Front Immunol. 2023 Nov 20;14:1293737. doi: 10.3389/fimmu.2023.1293737 (PMC10694275; doi:10.3389/fimmu.2023.1293737)

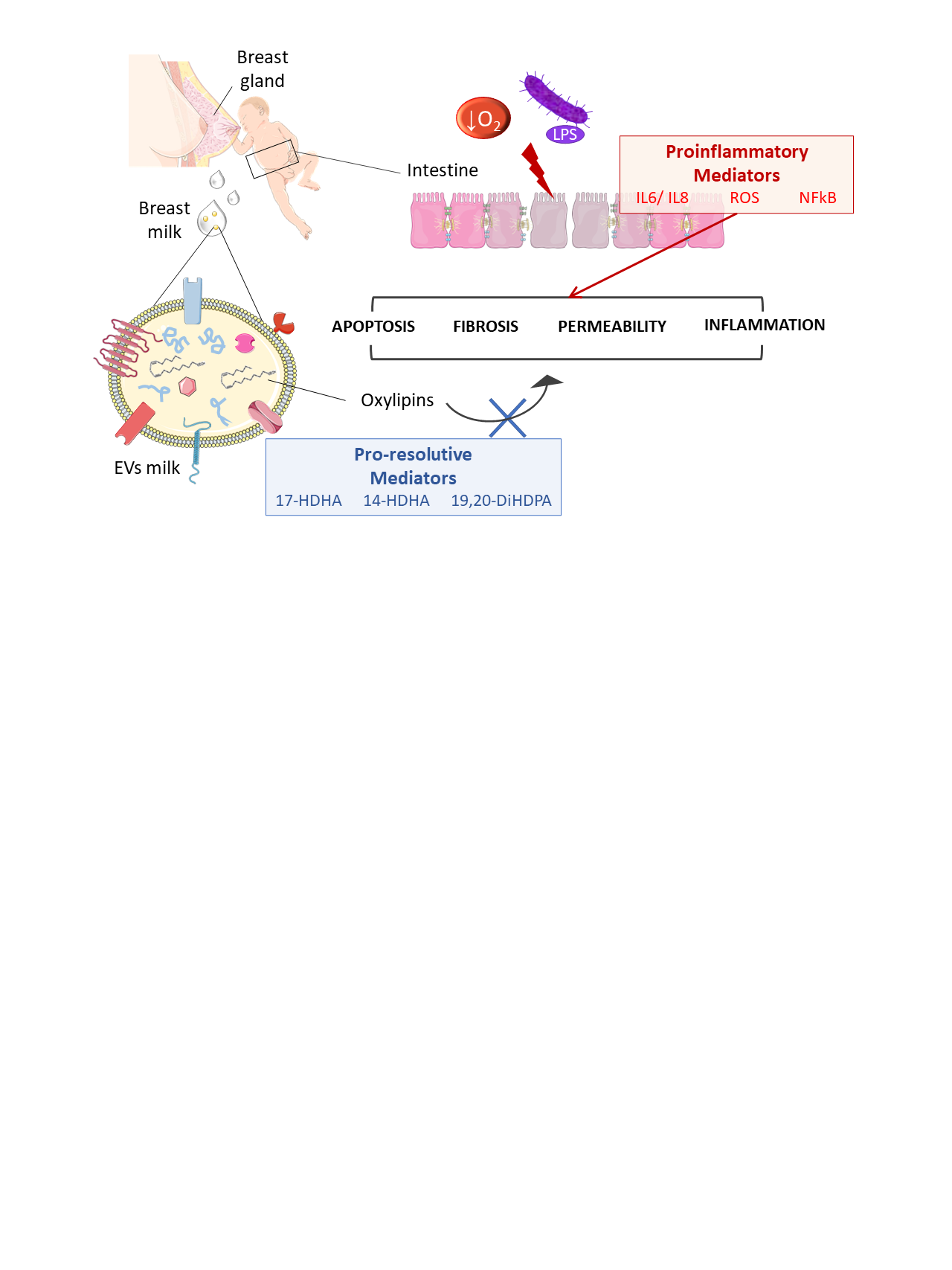

Supplement: Supplementary file 1 [file Image_1.tif]
